# Comparison of Unsupervised Home Self-collected Midnasal Swabs With Clinician-Collected Nasopharyngeal Swabs for Detection of SARS-CoV-2 Infection

**DOI:** 10.1001/jamanetworkopen.2020.16382

**Published:** 2020-07-22

**Authors:** Denise J. McCulloch, Ashley E. Kim, Naomi C. Wilcox, Jennifer K. Logue, Alex L. Greninger, Janet A. Englund, Helen Y. Chu

**Affiliations:** 1 Department of Medicine, Division of Allergy and Infectious Diseases, University of Washington, Seattle; 2 Department of Laboratory Medicine, University of Washington, Seattle; 3 Seattle Children’s Research Institute, Seattle, Washington

## Abstract

This diagnostic study compares unsupervised home self-collected midnasal swabs vs clinician-collected nasopharyngeal swabs for the detection of severe acute respiratory syndrome coronavirus 2 (SARS-CoV-2).

## Introduction

Increased diagnostics are urgently needed to contain the spread of coronavirus disease 2019 (COVID-19). Home self-collected swabs may increase testing access while minimizing exposure risk to health care workers and depletion of personal protective equipment, allowing for early community detection of COVID-19. A comparison of unsupervised home self-collected swabs with clinician-collected nasopharyngeal swabs for COVID-19 diagnosis has not been well described.

## Methods

This cross-sectional study was approved by the University of Washington institutional review board and follows the Strengthening the Reporting of Observational Studies in Epidemiology (STROBE) reporting guideline. Participants provided electronic informed consent. Study participants were recruited from symptomatic outpatients testing severe acute respiratory syndrome coronavirus 2 (SARS-CoV-2)–positive and symptomatic health care workers presenting to drive-through clinics (eFigure and eAppendix in the Supplement). Participants were provided test kits for unsupervised home self-collection of a midnasal swab. Home swab performance was compared with clinician-collected nasopharyngeal swabs, which were collected by medical assistants and nurses. Cycle thresholds (Ct) are a semiquantitative measure of viral load. Positive test results for SARS-CoV-2 by both approaches were defined as true positives. Results with a positive clinician swab and negative home swab were defined as false negatives. Sensitivity was defined as true positives divided by the sum of true positives and false negatives. Cohen κ was calculated for agreement between the 2 qualitative test results. The threshold for statistical significance was set at 2-tailed *P* < .05.

## Results

Of 185 total participants, 158 (85%) enrolled at drive-through clinics, and 27 (15%) enrolled after a positive SARS-CoV-2 test. Among the 185 participants, 41 (22.2%) yielded SARS-CoV-2 positive test results via clinician-collected nasopharyngeal swab, home self-collected midnasal swab, or both. One hundred fifty-eight participants (85%) were health care workers, of whom 14 (9%) tested positive. Among participants with COVID-19, common symptoms included myalgia (33 participants [80.5%]), cough (28 participants [68.3%]), and fever (26 participants [63.4%]). Compared with clinician swabs, sensitivity and specificity of home swabs was 80.0% (95% CI, 63%-91%) and 97.9% (95% CI, 94%-99.5%), respectively (Table). Cohen κ statistic was 0.81 (95% CI, 0.70-0.93), suggesting substantial agreement.

**Table.** Results of Clinician-Collected Nasopharyngeal Swabs vs Home Self-collected Midnasal Swabs

Self-collected MNS swab result	Median (IQR or range)			Total
	Clinician-collected NPS result			
	Positive	Negative	Inconclusive	
Positive, No.	28	3	0	31
NP swab viral load	24.1 (18.7-26.0)	NA	NA	24.1 (18.7-26.0)
Self-swab viral load	22.6 (19.1-27.3)	32.9 (32.7-33.2)	NA	22.8 (19.3-28.4)
Days between symptom onset and NP swab	3.0 (1.0-6.0)	4.0 (2.5-8.5)	NA	3.0 (1.0-6.0)
Days between MNS and NPS (range)	1.0 (0.0-2.0)	0.0 (0.0-0.0)	NA	1.0 (0.0-2.0)
Negative, No.	7	140	1	148
NP swab viral load	33.7 (33.5-35.1)	NA	37.4	34.4 (33.5-36.8)
Self-swab viral load	NA	NA	NA	NA
Days between symptom onset and NP swab	5.0 (2.5-13.0)	2.0 (1.0-4.0)	2.0	2.0 (1.0-4.0)
Days between MNS and NPS (range)	1.0 (0.0-1.0)	0.0 (−4.0 to 7.0)	0.0	0.0 (−4.0 to 7.0)
Inconclusive, No.^a^	3	3	0	6
NP swab viral load	32.9 (30.2-33.4)	NA	NA	32.9 (30.2-33.4)
Self-swab viral load	37.8 (37.3-37.9)	37.0 (37.0-37.0)	NA	37.4 (37.0-37.8)
Days between symptom onset and NP swab	5.5 (5.3-5.8)	1.5 (1.3-1.8)	NA	3.5 (1.8-5.3)
Days between MNS and NPS (range)	1.0 (1.0-1.0)	0.0 (0.0-0.0)	NA	0.5 (0.0-1.0)
Total, No.	38	146	1	185
NP swab viral load	24.5 (21.9-30.1)	NA	37.4	24.7 (22.3-31.8)
Self-swab viral load	22.9 (19.4-28.8)	33.4 (32.9-35.2)	NA	24.9 (19.9-32.9)
Days between symptom onset and NP swab	3.0 (2.0-6.0)	2.0 (1.0-4.0)	2.0	2.0 (1.0-5.0)
Days between MNS and NPS (range)	1.0 (0.0-2.0)	0.0 (−4.0 to 7.0)	0.0	0.0 (−4.0 to 7.0)

Abbreviations: IQR, interquartile range; MNS, midnasal swab; NA, not applicable; NPS, nasopharyngeal swab.

^a^ Positive result was defined as both of 2 primers positive, and inconclusive was defined as 1 of 2 probes for severe acute respiratory coronavirus 2 polymerase reaction positive and 1 negative.

Cycle thresholds of home swabs were positively correlated with clinician swabs (correlation coefficient, 0.81; *P* < .001) (Figure). Time from symptom onset to swab collection was comparable between true positives and false negatives. Among the 28 true positives, home swab collection occurred a median (interquartile range) of 4 (2-7) days after symptom onset, whereas among 7 false negatives, home swab collection occurred a median (interquartile range) of 6 (3-18) days after symptom onset (*P* = .32). The median (interquartile range) Ct of the clinician swab was lower for true positives vs false negatives (24.1 [18.7-26.0] vs 33.7 [33.5-35.1]; *P* = .01). Four of 5 false-negative swabs had Ct greater than or equal to 33. In a sensitivity analysis of all swabs with Ct less than or equal to 32, sensitivity of home swabs was 95%.

**Figure.**
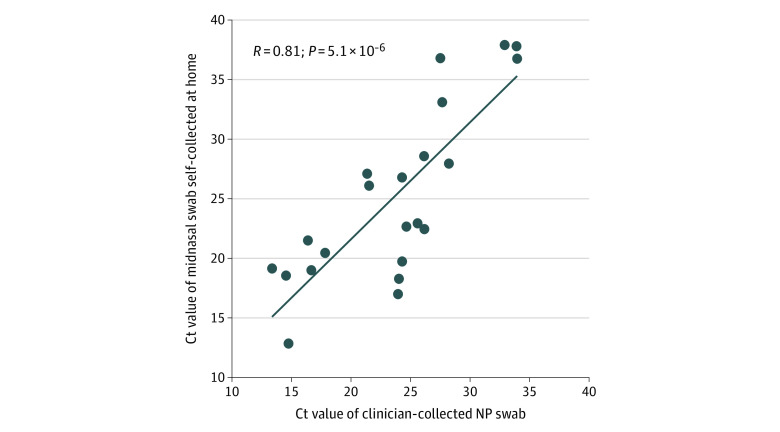
Cycle Thresholds (Ct) for Home Self-collected Midnasal Swabs and Clinician-Collected Nasopharyngeal (NP) Swabs Home self-collected midnasal swab (n = 28) Ct values were positively associated with the paired clinician-collected nasopharyngeal swab (n = 30) Ct value (correlation coefficient, 0.81; *P* = 5.1 × 10^−6^). The Ct values were calculated from a severe acute respiratory syndrome coronavirus 2 reverse transcriptase–polymerase chain reaction assay that targets 2 distinct regions of the virus, using Centers for Disease Control and Prevention primers and probes for the virus nucleocapsid (N) gene, N1 and N2.

## Discussion

Unsupervised home midnasal swab collection was comparable to clinician-collected nasopharyngeal swab collection for detection of SARS-CoV-2 in symptomatic patients, particularly those with higher viral loads. During this rapidly evolving pandemic, we enrolled 185 individuals presenting for SARS-CoV-2 testing, including 41 with positive test results. We used novel home-based swab self-collection and rapid delivery services, thus avoiding participant contact with the health care system.

Unsupervised home self-swab collection presents several advantages, including accessibility outside of the health care system and minimizing personal protective equipment use. This approach is safe and scalable in the pandemic setting, permitting widespread testing of symptomatic participants early in illness and the potential for prompt self-isolation and contract tracing. The sensitivity of home self-collection in this study was lower than previously described.^1^ We observed false-negative results in samples with low initial viral loads.^2,3,4^ A home-based strategy should be targeted toward individuals early in illness, when risk of transmission is highest and care seeking less likely.

Limitations of the study include shipping at ambient temperature, which may have led to sample degradation. However, we have demonstrated stability of respiratory viruses at ambient temperatures up to 9 days.^5^ Second, home self-collection often occurred 1 day after clinician collection, likely leading to samples with lower viral load. Third, many participants were health care workers, potentially limiting generalizability to the general population. Fourth, clinician-collected swabs are an imperfect criterion standard that may introduce bias.

As societies reopen, expansion of testing is critical for preventing a global resurgence in COVID-19. Home swab collection has the potential to play a pivotal role in increasing testing access across the broader population.

## Notes

Supplement. **eAppendix.** Supplemental methods **eReferences.** **eFigure.** Clinical study methods for enrollment of symptomatic healthcare workers presenting for drive-up testing and community outpatients with positive SARS-CoV-2 tests
