# Supplementary material for: Comparison of Unsupervised Home Self-collected Midnasal Swabs With Clinician-Collected Nasopharyngeal Swabs for Detection of SARS-CoV-2 Infection
Source: JAMA Netw Open. 2020 Jul 22;3(7):e2016382. doi: 10.1001/jamanetworkopen.2020.16382 (PMC7376392; doi:10.1001/jamanetworkopen.2020.16382)
Supplement: Supplement. — eAppendix. Supplemental methods eReferences. eFigure. Clinical study methods for enrollment of symptomatic healthcare workers presenting for drive-up testing and community outpatients with positive SARS-CoV-2 tests [file jamanetwopen-3-e2016382-s001.pdf]

Supplementary Online Content

McCulloch DJ, Kim AE, Wilcox NC, et al. Comparison of Unsupervised Home Self-collected Midnasal Swabs With Clinician-Collected Nasopharyngeal Swabs for Detection of SARS-CoV-2 Infection. *JAMA Netw Open*. 2020;3(7):e2016382. doi:10.1001/jamanetworkopen.2020.16382

eAppendix. Supplemental methods

eReferences

eFigure. Clinical study methods for enrollment of symptomatic healthcare workers presenting for drive-up testing and community outpatients with positive SARS-CoV-2 tests

This supplementary material has been provided by the authors to give readers additional information about their work.

© 2020 McCulloch DJ et al. *JAMA Network Open.*

eAppendix. Supplemental methods

Study Design
This was a cross-sectional study, with participants enrolled from March 31 through April 13, 2020.

Study Population
Study participants were recruited from two groups: (1) symptomatic outpatients presenting for care in the primary care system or emergency department (ED) who tested positive for SARS-CoV-2 and (2) symptomatic healthcare workers presenting to drive-through SARS-CoV-2 testing clinics at Harborview Medical Center and Northwest Hospital, all at the University of Washington in Seattle, WA, USA.

For the first group, the research team received notification of potential participants with a positive SARS-CoV-2 test result based on clinical testing through an electronic alert from the clinical laboratory. They were subsequently contacted by the research team, underwent informed consent over the phone, and signed an electronic consent form using Project REDCap.[1] Participants subsequently had a home swab kit delivered to their home within hours. In the second group, symptomatic healthcare workers presenting to SARS-CoV-2 drive-through testing clinics had the option to sign up to obtain a home swab kit at the time of testing while making their drive-through clinic appointment. This kit contained a link to a web portal with information about the study and a secure electronic consent form on REDCap. Participants reviewed study information electronically and signed an online consent form. The planned sample size was approximately 200 participants, including about 40 COVID-19 positive individuals. These numbers reflect what was realistically possible during the pandemic, while attempting to capture sufficient numbers to accurately estimate sensitivity and specificity.

Following consent, both groups completed an online questionnaire to assess demographics, symptoms, medical comorbidities, and care-seeking behavior. All participants received a home swab collection kit containing a flocked mid-nasal swab (Copan FloqSwab 56380CS01, Copan Diagnostics, Murrieta, CA), Universal Transport Media (UTM) (Becton Dickinson, Franklin, NJ), and written instructions on how to perform a self-collected nasal swab.[2,3] Following home swab collection, swabs were returned to the lab via either same-day courier or via USPS, following standard IATA shipping procedures.[4] Samples were subsequently aliquoted and transferred to the University of Washington Clinical Virology Laboratory at room temperature, and stored at 4°C prior to testing.

Laboratory Methods
RT-PCR for detection of SARS-CoV-2 was performed using a test developed by the UW Clinical Virology laboratory, as previously described.[5,6] Briefly, 200µL of the viral media from nasal/nasopharyngeal swabs collected in 3mL of viral transport is extracted on a high-throughput Roche Magna Pure 96 using the Viral NA small volume kit and eluted into 50µL of elution buffer. Next, 5µL of eluate was used as a template for a one-step RT-PCR performed using 25µL total reaction with AgPath-ID system master mix on the real-time ABI 7500 instrument. The Real-Time RT PCR assay targets two distinct regions of SARS-CoV-2, using Centers for Disease Control (CDC) primers and probes for the virus nucleocapsid (N) gene, N1 and N2. Amplification of both N-gene targets results in a positive result, amplification of one results in an inconclusive result, and amplification of neither results in a negative result.

Data analysis
Data were analyzed in R version 3.6.0 (Vienna, Austria) and SAS 9.4 (Cary, NC). Chi-square tests, p values, and 95% confidence intervals (CIs) were calculated for categorical variables. Wilcoxon rank-sum tests were used to compare participants whose swabs were both negative for SARS-CoV-2 to those who had any positive. Wilcoxon signed-rank tests were calculated to compare average CT values from the home mid-nasal swabs versus the clinician-collected NP swab within participants who had detection of SARS-CoV-2 (Table 1).

False negative tests were defined as a negative home mid-nasal swab and a positive clinician-collected NP swab. True positives were defined as a positive result on both the home mid-nasal swab and the clinician-collected NP swab. A sensitivity analysis was performed among a subset of 23 samples with a NP swab with Ct $\leq$32.

Ct values were unavailable for a subset of participants (24% were missing the Ct value for the NP swab, and 18% were missing the Ct value for the self-swab.) Missing Ct values were omitted from the analysis.

© 2020 McCulloch DJ et al. *JAMA Network Open.*

eReferences

1. (REDCap)--a metadata-driven methodology and workflow process for providing translational research informatics support. *J Biomed Inform.* 2009;42(2):377-381.
2. Chu HY, Englund JA, Starita LM, et al. Early Detection of Covid-19 through a Citywide Pandemic Surveillance Platform. *New England Journal of Medicine.* 2020.
3. Kim AE, Brandstetter E, Graham C, et al. Seattle Flu Study - Swab and Send: Study Protocol for At-Home Surveillance Methods to Estimate the Burden of Respiratory Pathogens on a City-Wide Scale. *medRxiv.* 2020:2020.2003.2004.20031211.
4. IATA. *Dangerous goods regulations manual.* 2020.
5. Nalla AK, Casto AM, Huang M-LW, et al. Comparative Performance of SARS-CoV-2 Detection Assays Using Seven Different Primer-Probe Sets and One Assay Kit. *Journal of Clinical Microbiology.* 2020;58(6):e00557-00520.
6. Bhatraju PK, Ghassemieh BJ, Nichols M, et al. Covid-19 in Critically Ill Patients in the Seattle Region - Case Series. *N Engl J Med.* 2020.

© 2020 McCulloch DJ et al. *JAMA Network Open.*

eFigure. Clinical study methods for enrollment of symptomatic healthcare workers presenting for drive-up testing and community outpatients with positive SARS-CoV-2 tests

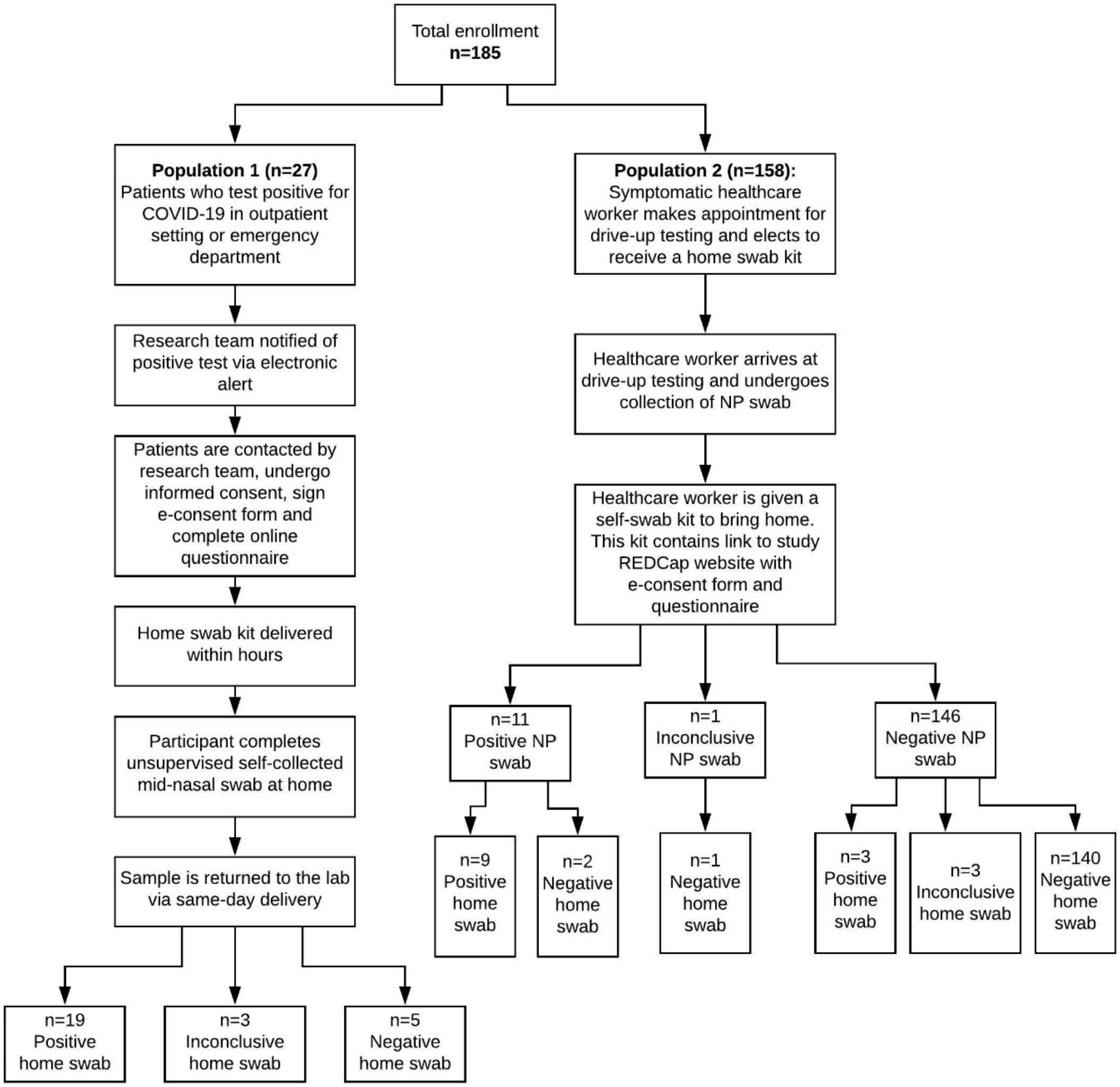

© 2020 McCulloch DJ et al. *JAMA Network Open.*
